# Treatment-related toxicity using prostate bed versus prostate bed and pelvic lymph node radiation therapy following radical prostatectomy: A national population-based study

**DOI:** 10.1016/j.ctro.2023.100622

**Published:** 2023-04-11

**Authors:** Arunan Sujenthiran, Matthew G. Parry, Joanna Dodkins, Julie Nossiter, Melanie Morris, Brendan Berry, Arjun Nathan, Paul Cathcart, Noel W. Clarke, Heather Payne, Jan van der Meulen, Ajay Aggarwal

**Affiliations:** a Clinical Effectiveness Unit, Royal College of Surgeons of England, UK; b Department of Health Services Research & Policy, LHSTM, UK; c Department of Radiotherapy, Guy’s & St Thomas’ NHS Foundation Trust, UK; d Department of Cancer Epidemiology, Population & Global Health, KCL, UK; e Flatiron Health, UK; f Department of Oncology, University College London Hospitals, London, UK; g Department of Urology, Guy’s & St Thomas’ NHS Foundation Trust, UK; h Department of Urology, The Christie & Salford Royal NHS Foundation Trusts, UK

**Keywords:** Pelvic lymph node irradiation, Prostate-bed only radiation therapy, Gastrointestinal, Genitourinary toxicity

## Abstract

Purpose There is debate about the effectiveness and toxicity of pelvic lymph node (PLN) irradiation in addition to prostate bed radiotherapy when used to treat disease recurrence following radical prostatectomy. We compared toxicity from radiation therapy (RT) to the prostate bed and pelvic lymph nodes (PBPLN-RT) with prostatebed only radiation therapy (PBO-RT) following radical prostatectomy. Methods and Materials Patients with prostate cancer who underwent post-prostatectomy RT between 2010 and 2016 were identified by using the National Prostate Cancer Audit (NPCA) database. Follow-up data was available up to December 31, 2018. Validated outcome measures, based on a framework of procedural and diagnostic codes, were used to capture ≥Grade 2 gastrointestinal (GI) and genitourinary (GU) toxicity. An adjusted competing-risks regression analysis estimated subdistribution hazard ratios (sHR). A sHR > 1 indicated a higher incidence of toxicity with PBPLN-RT than with PBO-RT. Results 5-year cumulative incidences in the PBO-RT (n = 5,087) and PBPLNRT (n = 593) groups was 18.2% and 15.9% for GI toxicity, respectively. For GU toxicity it was 19.1% and 20.7%, respectively. There was no evidence of difference in GI or GU toxicity after adjustment between PBO-RT and PBPLN-RT (GI: adjusted sHR, 0.90, 95% CI, 0.67–1.19; P = 0.45); (GU: adjusted sHR, 1.19, 95% CI, 0.99–1.44; P = 0.09). Conclusions This national population-based study found that including PLNs in the radiation field following radical prostatectomy is not associated with a significant increase in rates of ≥Grade 2 GI or GU toxicity at 5 years.

## Highlights

• Limited evidence on toxicity of pelvic lymph node plus prostate bed radiotherapy.
• This is the largest observational study assessing toxicity rates in this setting.
• There is no significant increase in genitourinary or gastrointestinal toxicity.
• This treatment should be considered in patients at a high risk of nodal relapse.

## Introduction

External beam radiotherapy (RT) to the prostate bed is currently a standard treatment for men with PSA failure after radical prostatectomy. Recent evidence from randomized controlled trials (RCTs) [1], [2] has supported an initial observation policy following surgery, with salvage RT recommended for those with confirmed biochemical recurrence (PSA ≥ 0.1). However, adjuvant radiation therapy may be indicated [3] in men with high-risk (N1 or Gleason score 8 to 10 and T3/4) prostate cancer. Whilst salvage radiotherapy is an option for men with PSA failure after radical prostatectomy, there remains uncertainty regarding whether to target the pelvic lymph nodes (PLNs) in the salvage radiation field, with conflicting results from existing studies [4], [5], [6], [7] and clinical guidelines not demonstrating a consensus [8]. The rationale for including the PLNs as well as the prostate bed is supported by different studies including data from the EMPaCT group [9] that demonstrated a high rate of microscopic lymph node disease, particularly in patients with high-risk prostate cancer. Also, PSMA PET-CT studies in men with PSA failure after radical prostatectomy, have reported that the PLNs are a common site of post-surgical recurrent disease [10]. Although PSMA PET-CT has reduced sensitivity at low PSA levels, studies using this imaging modality have been used to recently validate the recent changes to radiotherapy planning guidance to ensure coverage of at risk areas [11]. At present, the decision regarding treatment post prostatectomy should be based on evidence for biochemical relapse and not on waiting for evidence of visible relapse on PSMA PET-CT. Furthermore, recent RCT evidence from the NRG Oncology/RTOG 0534 SPPORT trial in men receiving salvage post-prostatectomy RT reported demonstrating an improvement in biochemical control for whole pelvis versus prostate-bed only RT [12].

However, concerns remain about increased genitourinary (GU) and gastrointestinal (GI) treatment-related toxicity in patients receiving pelvic lymph node RT due to the increased irradiated volume. In this study we use data from the National Prostate Cancer Audit (NPCA), which collects information on all newly diagnosed patients in the English NHS (covering 95% of the eligible population). Within this we use administrative datasets, cancer registry and radiotherapy treatment specific information to evaluate how prostate bed and pelvic lymph node radiotherapy (PBPLN-RT) impacts treatment-related toxicity in comparison to those who underwent prostate-bed only radiation therapy (PBO-RT) using previously validated indicators [13].

## Materials (patients) and methods

### Patient population

In this study we used the National Prostate Cancer Audit (NPCA) database which includes data linked at a patient-level from the English Cancer Registry [14], the National Radiotherapy Dataset (RTDS) [15] and Hospital Episode Statistics (HES) [16]. We identified men with a diagnosis of prostate cancer and treated with post-prostatectomy RT between January 1, 2010 and December 31, 2016.

### Inclusion and exclusion criteria

**Fig. 1:**
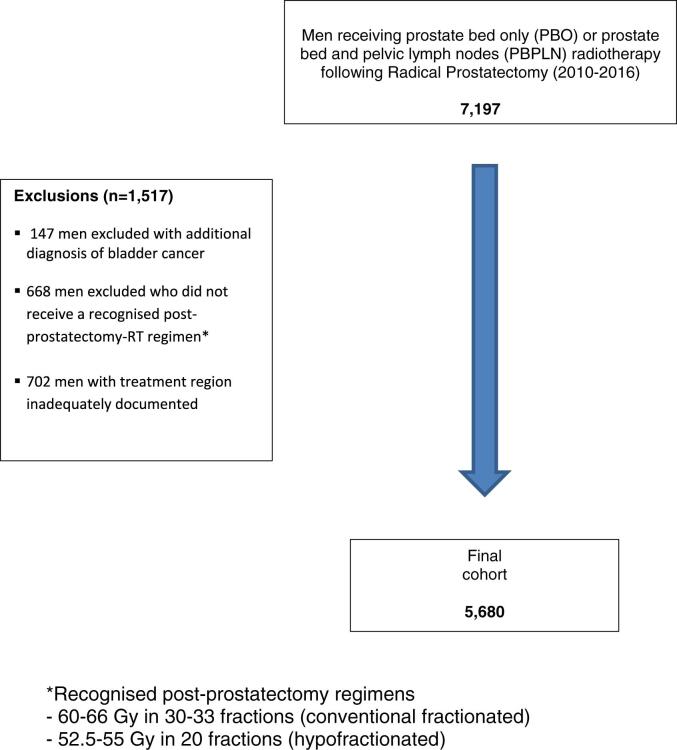
Flow chart of men included in study. *Recognized post-prostatectomy regimens. – 60-66 Gy in 30-33 fractions (conventional fractionated). – 52.5-55 Gy in 20 fractions (hypofractionated).

The records of 7,197 men from the NPCA who had non-metastatic prostate cancer who received post-prostatectomy RT were studied. Patients were excluded with an associated bladder cancer diagnosis (ICD-10 “C67”) (n = 147), when the RT treatment region was not recorded (n = 702) and if they did not receive recognized post-prostatectomy regimens (60–66 Gy in 30–33 fractions (conventional); 52.5–55 Gy in 20 fractions (hypofractionated)) in concordance with UK radiotherapy guidance and/or regimens used in randomized controlled trials (n = 668) [17], [18], [19], [20]. The final cohort included 5,680 men (Fig. 1).

### Study outcome

We used previously validated performance indicators to identify men who experienced urinary or bowel-related toxicity that required a diagnostic or therapeutic procedure following radiotherapy [21]. This was based on an established framework where procedures are coded using the UK Office for Population Census and Surveys classification, 4th revision (OPCS-4) [22], and the diagnosis codes labelled using the International classification of Diseases, 10th revision (ICD-10) [23]. Men were classified as having experienced a complication if both a diagnostic procedure and corresponding diagnosis code for radiation-related GI or GU toxicity could be identified in a patient record following the RT treatment start date. Consequently, our approach limited our analyses to toxicity of at least grade 2 according to the National Cancer Institute Common Toxicity Criteria for Adverse Events (moderate severity requiring local or non-invasive interventions) [24].

The RTDS provided information on the use of an intensity-modulated radiotherapy (IMRT) technique (OPCS-4 code “X671”) and the RT field (PBO and PBPLN). It was acknowledged that we could only ascertain the total PBPLN dose and not isolated doses delivered to the lymph nodes. RTDS doses and attendances were also used to ascertain whether a patient received a conventional or hypofractionated regimen.

Data items in HES records were used to determine age, the Royal College of Surgeons (RCS) Charlson comorbidity score (expressed as the number of comorbidities) [25] and socioeconomic deprivation status according to quintiles of the national ranking of the Index of Multiple Deprivation [26]. HES records were also used to identify the type of radical prostatectomy (open, laparoscopic and robot-assisted) and whether a pelvic lymph node dissection was performed at the time of radical prostatectomy, using a coding framework previously described [27]. Tumor characteristics (T-stage, N-stage and Gleason score) were obtained from the linked cancer registry records and then used to determine the disease risk-classification with a previously described NPCA algorithm [28].

The baseline GI and GU function of the patients included was estimated based on the presence of a GI or GU procedure code in the HES record up to one year before the start of RT. This was used as a proxy of whether patients had significant bowel or urinary issues prior to treatment, as previously described [13].

Information on the decision to trigger post RP RT (i.e. adjuvant vs salvage approach) was not available. Therefore, we stratified men into those who started RT within 6 months of RP and those who started after 6 months. This was in line with protocols used in existing RCTs assessing timing of post-prostatectomy RT [17], [29].

### Statistical analysis

We calculated the cumulative incidence of GI and GU toxicity from initiation of RT until cessation of follow-up at a maximum of five years. Death from any cause was considered a competing event [30]. Fine and Gray competing risk regression analysis estimated sub distribution hazard ratios (sHR) with 95% confidence intervals (CIs), comparing the risk of GU and of GI toxicity events between PBPLN-RT and PBO-RT groups [31]. Patients were censored at the end of follow-up and the regression analysis was adjusted for cohort demographics. Missing data points for cancer risk profile (n = 811) were imputed using multiple imputation by chained equations. In all, 50 data sets were created, and Rubin’s rules were used to combine the sHRs. Wald tests were used to calculate P values with significance set at P < 0.05.

## Results

### Patient population

Table 1 presents the study cohort’s characteristics (n = 5,680). The median age (interquartile range) of the cohort was 65 (61–69) years and 68.3% of men had locally advanced/high risk disease and 29.3% received radiotherapy within 6 months of radical prostatectomy. The majority of patients received a conventional radiotherapy regimen (72.4% in the PBO group and 95.8% in the PBPLN-RT group).

**Table 1:** Patient, disease and treatment of characteristics of men receiving post-prostatectomy radiotherapy.

Characteristic	PBO RT (N= 5,087)		PBPLN RT (N= 593)		All patients (N= 5,680)	
	No.	%	No.	%	No.	%
Treatment year						
2010	348	6.8	16	2.7	364	6.4
2011	431	8.5	34	5.7	465	8.2
2012	699	13.7	43	7.3	742	13.1
2013	749	14.7	101	17.0	850	15.0
2014	808	15.9	140	23.6	948	16.7
2015	1,021	20.1	126	21.2	1,147	20.2
2016	1,031	20.3	133	22.4	1,164	20.5

Age group, years						
<60	1,069	21.0	137	23.1	1,206	21.2
60-70	2,902	57.0	351	59.2	3,253	57.3
>70	1,116	21.9	105	17.7	1,221	21.5

No. of comorbidities (RCS Charlson score)						
0	4,364	85.8	478	80.6	4,842	85.2
1	622	12.2	98	16.5	720	12.7
≥2	101	2.0	17	2.9	118	2.1

Deprivation status (national quintiles)						
1(least deprived)	1,301	25.6	145	24.5	1,446	25.5
2	1,264	24.8	131	22.1	1,395	24.6
3	1,100	21.6	129	21.8	1,229	21.6
4	836	16.4	115	19.4	951	16.7
5(most deprived)	586	11.5	73	12.3	659	11.6

Risk classification group						
Locally advanced/high risk	2,903	67.1	424	78.4	3,327	68.3
Intermediate	1,307	30.2	112	20.7	1,419	29.1
Low risk	118	2.7	5	0.9	123	2.5
Unclassifiable	759	14.9	52	8.8	811	14.3

GI procedure 1 year before RT						
0	4,923	96.8	575	97.0	5,498	96.8
1	164	3.2	18	3.0	182	3.2

GU procedure 1 year before RT						
0	4,836	95.1	568	95.8	5,404	95.1
1	251	4.9	25	4.2	276	4.9

Type of radical prostatectomy						
Robotic	2,141	42.1	311	52.4	2,452	43.2
Laparoscopic	1,335	26.2	109	18.4	1,444	25.4
Open	1,611	31.7	173	29.2	1,784	31.4

Pelvic lymph node dissection performed						
No	3,210	63.1	313	52.8	3,523	62.0
Yes	1,877	36.9	280	47.2	2,157	38.0

RT technique						
3D conformal	2,154	42.3	113	19.1	2,267	39.9
IMRT	2,933	57.7	480	80.9	3,413	60.1

Type of RT regimen						
Conventional	3,684	72.4	568	95.8	4,252	74.9
Hypofractionated	1,403	27.6	25	4.2	1,428	25.1

Time between RP and RT						
<6 months	1,494	29.4	171	28.8	1,665	29.3
≥6 months	3,593	70.6	422	71.2	4,015	70.7

Men who received PBPLN-RT were more likely to have had locally advanced disease, undergone robotic radical prostatectomy with a concomitant pelvic lymph node dissection and received intensity-modulated radiotherapy (IMRT) (Table 1). The PBO-RT group had a higher proportion of men in the age category > 70 years old. The Charlson comorbidity scores and estimated baseline GI/GU toxicity (based on occurrence of GI/GU procedures in the 1 year prior to RT) were similar between study groups.

The doses and fractionation schedules for the PBO group were 66 Gy in 33 fractions (62.3%), 64 Gy in 32 fractions (5.8%), 55 Gy in 20 fractions (5.1%), 52.5 Gy in 20 fractions (21.5%) and other (5.3%). The doses and fractionation schedules for the PBPLN group were 66 Gy in 33 fractions (86.3%), 64 Gy in 32 fractions (1.7%), 55 Gy in 20 fractions (0.8%), 52.5 Gy in 20 fractions (3.0%) and other (8.1%). The median follow up (interquartile range) was similar for both groups, with 4.3 (3.1–5.9) years for the PBO group and 4.2 (3.0–5.2) years for the PBPLN group.

### Outcome measures

**Fig. 2:**
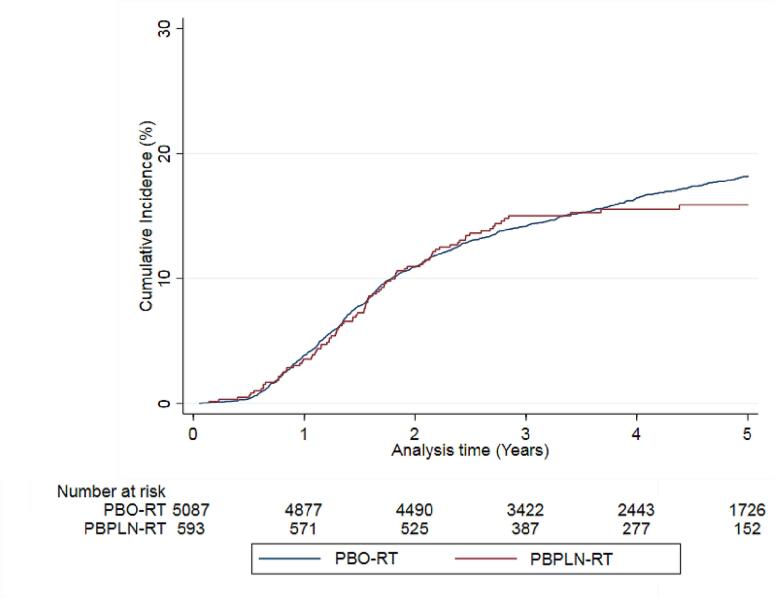
Cumulative incidence curves for gastrointestinal toxicity after post-prostatectomy radiotherapy to the prostate bed only (PBO-RT) or the prostate bed and pelvic lymph nodes (PBPLN RT).

**Fig. 3:**
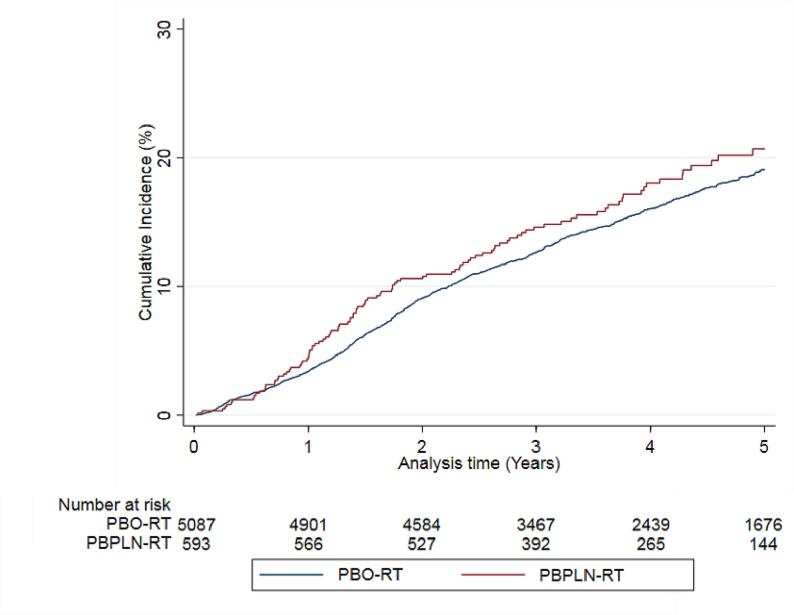
Cumulative incidence curves for genitourinary toxicity after post-prostatectomy radiotherapy to the prostate bed only (PBO-RT) or the prostate bed and pelvic lymph nodes (PBPLN-RT).

The 5-year cumulative incidence of GI toxicity was 15.9% (95 %CI 13.0 to 19.1) in the PBPLN-RT group and 18.2% (95 %CI 17.1 to 19.4) in the PBO-RT group. The 5-year cumulative incidence of GU toxicity was 20.7% (95 %CI 17.1 to 24.6) in the PBPLN-RT groups and 19.1% (95 %CI 17.9 to 20.3) in the PBO-RT group (Fig. 2, Fig. 3, Table 2).

**Table 2:** Adjusted outcomes for GI and GU toxicity after PBO-RT or PBPLN-RT.

Toxicity site	5-year cumulative incidence (%)	95% CI	sHR*	95% CI	P
GI toxicity					
PBO-RT	18.2	17.1–19.4	1		
PBPLN-RT	15.9	13.0–19.1	0.90	0.67–1.19	0.45

GU toxicity					
PBO-RT	19.1	17.9–20.3	1		
PBPLN-RT	20.7	17.1–24.6	1.19	0.99–1.44	0.07

*Adjusted for treatment year, age group, RCS Charlson score, deprivation status, risk classification group, GI procedure 1 year before RT, GU procedure 1 year before RT, type of radical prostatectomy, pelvic lymph node dissection performed, RT technique, type of RT regimen, time between radical prostatectomy and RT.

Applying a competing risks approach with adjustment resulted in no statistically significant difference in GI toxicity (adjusted sHR, 0.90, 95% CI, 0.67 to 1.19; P = 0.45) or in GU toxicity (adjusted sHR, 1.19, 95% CI, 0.99 to 1.44; P = 0.09) between both groups (Table 2). There was no association between toxicity and age group, deprivation status, risk classification group, previous GI procedure 1 year before RT, type of RP performed, PLND performed, use of IMRT, use of hypofractionation and time between RP and RT (Appendix 1 and 2).

Men with a higher comorbidity score experienced higher rates of GI and GU toxicity and those who underwent a GU procedure 1 year prior to RT had greater GU toxicity. Patients receiving hypofractionated radiotherapy had a lower risk of GI toxicity (Appendix 1 and 2).

A sensitivity analysis was performed for the patients who received a conventional radiotherapy regimen (PBO-RT: 3684 patients; PBPLN-RT: 568 patients). The 5-year cumulative incidence of GI toxicity increased from 15.9% to 16.6% (95 %CI 13.6 to 19.9) in the PBPLN-RT group and 18.2% to 20.0% (95 %CI 18.6 to 21.4) in the PBO-RT group, but differences remained statistically insignificant (adjusted sHR, 0.96, 95% CI, 0.72 to 1.28; P = 0.78).

However, the 5-year cumulative incidence of GU toxicity increased slightly from 20.7% to 21.0% (95 %CI 17.2 to 25.0) in the PBPLN-RT group and remained at 19.1% (95 %CI 17.9 to 20.3) in the PBO-RT group and the differences became statistically significant (adjusted sHR, 1.22, 95% CI, 1.01 to 1.47; P = 0.04) between both groups.

## Discussion

Our study of 5,680 men undergoing post-prostatectomy radiotherapy did not find a statistically significant difference in GI toxicity for men receiving RT to the prostate bed with or without pelvic lymph-node irradiation. There was a trend towards increased GU toxicity in the PBPLN-RT group but this was not statistically significant after adjustment for patient and tumor characteristics. We also found that having significant comorbidities was associated with increased GI and GU toxicity and that patients who had previous GU interventions had an increased likelihood of GU toxicity following treatment. We also found that the use of hypofractionated RT was associated with lower rates of GI toxicity.

Evidence from RCTs and observational studies comparing rates of toxicity between men receiving pelvic lymph node irradiation with prostate bed radiotherapy is limited. The NRG Oncology/RTOG 0534 SPPORT RCT (median follow-up 8.2 years) [12] reported data showing late grade 3 + GU toxicity: in PBO-RT and PBPLN-RT it was 5% and 8%, respectively, Grade 3 + GI toxicity was 1% and 1%, respectively. There were no significant differences between the groups in late grade 2 or worse or grade 3 or worse GU or GI adverse events. Our results in a large national cohort including all patients treated with this approach correlate with these RCT findings.

Previous observational studies evaluating toxicity report conflicting results. One study included 742 men undergoing post-prostatectomy RT from a single center from 1993 to 2005. There was a significantly higher 8-year incidence of late Grade 3 GU toxicity (16% in the whole-pelvis group versus 11% in the prostate-bed only group). In contrast, another single-institution dosimetric study compared prostate-bed only and whole-pelvis post-prostatectomy IMRT in 67 men treated between 2006 and 2009, with a 25 month median follow-up. They demonstrated no differences in acute/late GU or late GI toxicity despite higher dosimetric values for irradiated bowel, bladder and rectum in the whole-pelvis group [32].

The impact of elective irradiation of the PLNs on rates of GI and GU toxicity in the primary prostate radiotherapy setting is unclear with two RCTs reporting different results. The RTOG-9413 trial showed that prostate and PLN RT compared to prostate-only RT was associated with an increase in acute Grade 2 GI and GU toxicity, and late Grade 3 GI toxicity according to the RTOG scale [33]. In contrast the GETUG-01 RCT observed similar GI and GU toxicity between groups [34]. Previous results from a study, using NPCA data, were in concordance with findings from the GETUG-01 RCT and did not demonstrate an increased toxicity with additional pelvic nodal RT in the primary setting [13].

The current study has a number of strengths. First, to our knowledge, this is the largest comparative study assessing the effect of additional post-prostatectomy PLN RT on toxicity. In conjunction with the NRG Oncology/RTOG 0534 SPPORT trial, our study provides important comparative data on toxicity for patients and clinicians. Furthermore using observational data to capture adverse events can provide a more accurate representation of the frequency of toxicity compared with RCT populations, which can often result in underestimation [35].

Second, our findings are representative of real-world practice on a national scale in a publicly funded national health-care system, treated in centralized, high treatment-volume departments. Patients undergoing RT in private hospitals are not captured in the NPCA database, representing approximately less than 10% of cases in England.

Third, through linkage with RTDS, we extracted detailed information regarding RT doses and patient attendances. As a result we only included men who received recognized post-prostatectomy regimens (conventional and hypofractionated).

Finally, the indicators we used have been specifically developed and validated to capture radiotherapy-related toxicity requiring admission or an intervention that allowed us to measure GI and GU toxicity at a specific severity level [13], [20], [21]. Although we did not have information on baseline bowel/urinary function, we have accounted for differences by adjusting for GI and GU procedures in the previous 12 months, which has been defined in previous studies [13].

There are some limitations to this study. Information on the use of hormonal therapy was not available, however results from previous studies demonstrated its use is not associated with GI and GU toxicity [36], [37]. The sample size for the pelvic nodal group is small and we were not able to capture the extent of PLN performed at time of RP. Data related to the intent of RT treatment (adjuvant vs salvage) was not available, but this would unlikely affect our outcome measure of toxicity as we used the “time between RP and RT” as an alternative data point.

Recent research has investigated the role of IMRT in reducing the risk of GI and GU toxicity, after radical prostatectomy. A study by Alongi and colleagues suggested that the use of IMRT may reduce toxicity in the post-prostatectomy setting [38]. In this study 172 patients received PBPLN, with 81 patients receiving three-dimensional conformal and 91 patients receiving IMRT. The patients treated with IMRT experienced a decreased risk of acute toxicity (grade > or = 2 toxicity was GU 12.3% vs. 6.6% (p = 0.19); GI 8.6% vs. 3.2% (p = 0.14); for 3DCRT and IMRT, respectively). In the current study, a higher proportion of patients received IMRT in the PBO group compared to PBPLN group (80% versus 60%, respectively). IMRT was not associated with increased toxicity in the multivariable analysis (Appendix 1 and 2). This is consistent with a previous NPCA study which found no difference in GU and GI toxicity between patients receiving 3D-conformal RT vs IMRT in the post-prostatectomy setting [20].

The RTDS captures the total dose delivered to the prostate bed and lymph nodes as opposed to specific boosts delivered to the lymph nodes. As a result, we cannot account for the variation in boost doses received in the PBPLN cohort but this should not affect the comparison between the PBPLN and PBO groups. Whilst we utilised a validated outcome measure to define toxicity, our coding framework did not identify patients who experience milder Grade 1 toxicity in isolation, which can also impact a patient’s quality of life.

Although the evidence for the routine use of PBPLN-RT in terms of oncological benefit remains uncertain, many groups believe that the PLNs should be targeted. In contrast other groups refrain from offering this therapy due to concerns about augmented toxicity. Our national population-based study, using a previously validated toxicity indicator, has demonstrated that targeting the PLNs in the post-prostatectomy setting is not associated with a significant increase in rates of ≥ Grade 2 GI or GU toxicity at 5 years. This data together with emerging findings from studies including the NRG Oncology/RTOG 0534 SPPORT trial, EMPACT database and other ongoing trials incorporating the use of modern imaging techniques such as PSMA PET-CT will help provide further evidence to clinicians and patients in this area [12], [39].

## Conclusion

This national population-based study has shown that targeting the PLNs in the radiation field following radical prostatectomy is not associated with a significant increase in rates of ≥ Grade 2 GI or GU toxicity at 5 years.

A.S. is an employee of Flatiron Health, an independent subsidiary of the Roche group, and holds stock in Roche. H.P. has attended and received honoraria for advisory boards, travel expenses to medical meetings, and served as a consultant for AstraZeneca, Astellas, Janssen, Sanofi Aventis, Takeda, Ipsen, Ferring, Sandoz, and Novartis. N.W.C. has attended and received honoraria for advisory boards, travel expenses to medical meetings, and served as a consultant for AstraZeneca, Astellas, Bayer, Janssen, Sanofi Aventis, Takeda, Ipsen and Ferring. J.v.d.M. reports a contract with the Healthcare Quality Improvement Partnership for the provision of the National Prostate Cancer Audit (www.npca.org,uk) funded by the Healthcare Quality Improvement Partnership (www.hqip.org.uk).

## Funding

M.G.P. was supported by the National Institute of Health Research (DRF-2018–11-ST2-036. B.B. was partly supported by the NHS National Institute for Health Research through an Academic Clinical Fellowship. AA was supported by a National Institute for Health Research Advanced Fellowship (NIHR300599), H.P. was supported by the University College London Hospitals/University College London Comprehensive Biomedical Research Centre. J.v.d.M. was partly supported by the National Institute for Health Research Collaboration for Leadership in Applied Health Research and Care North Thames at Bart’s Health NHS Trust. The views expressed in this article are those of the authors and not necessarily those of the NHS, the National Institute for Health Research or the Department of Health and Social Care.

## Ethics approval

All patient data used is fully anonymised and is therefore exempt from UK National Research Ethics Committee (NREC) approval.

## Data and materials/data sharing policy

The cancer registry data used for this study are based on information collected and quality assured by Public Health England’s National Cancer Registration Service (www.ncras.nhs.uk). Access to the data was facilitated by the Public Health England’s Office for Data Release. Hospital Episode Statistics were made available by the NHS Digital (www.digital.nhs.uk); all rights reserved). M.G.P. had full access to all the data in the study and takes responsibility for the integrity of the data and accuracy of the data analysis. Data are not available to other researchers as it uses a registry database of patients providing routinely collected data.

## Declaration of Competing Interest

The authors declare the following financial interests/personal relationships which may be considered as potential competing interests: A.S. is an employee of Flatiron Health, an independent subsidiary of the Roche group, and holds stock in Roche. H.P. has attended and received honoraria for advisory boards, travel expenses to medical meetings, and served as a consultant for AstraZeneca, Astellas, Janssen, Sanofi Aventis, Takeda, Ipsen, Ferring, Sandoz, and Novartis. N.W.C. has attended and received honoraria for advisory boards, travel expenses to medical meetings, and served as a consultant for AstraZeneca, Astellas, Bayer, Janssen, Sanofi Aventis, Takeda, Ipsen and Ferring. J.v.d.M. reports a contract with the Healthcare Quality Improvement Partnership for the provision of the National Prostate Cancer Audit (www.npca.org.uk) funded by the Healthcare Quality Improvement Partnership (www.hqip.org.uk).
