# Supplementary material for: Treatment-related toxicity using prostate bed versus prostate bed and pelvic lymph node radiation therapy following radical prostatectomy: A national population-based study
Source: Clin Transl Radiat Oncol. 2023 Apr 11;40:100622. doi: 10.1016/j.ctro.2023.100622 (PMC10159812; doi:10.1016/j.ctro.2023.100622)
Supplement: Supplementary data 1 [file mmc1.docx]

**APPENDIX**

**Table 1.** Multivariable analysis of factors associated with developing gastrointestinal (GI) toxicity following prostate bed and pelvic lymph node radiotherapy (PBPLN).

|  | **Adjusted sHR^*^** | **95% Confidence Interval** |  | |  | ***p*** |
| --- | --- | --- | --- | --- | --- | --- |
| **Radiotherapy treatment region** | |  |  | |  | 0.45 |
| PBO | 1 |  |  | |  |  |
| PBPLN | 0.90 | 0.67 | - | | 1.19 |  |
|  |  |  |  | |  |  |
| **Treatment year** |  |  |  | |  | 0.69 |
| 2010 | 1 |  |  |  | |  |
| 2011 | 0.98 | 0.73 | - | 1.30 | |  |
| 2012 | 1.02 | 0.75 | - | 1.40 | |  |
| 2013 | 1.00 | 0.76 | - | 1.31 | |  |
| 2014 | 0.89 | 0.67 | - | 1.19 | |  |
| 2015 | 0.91 | 0.67 | - | 1.22 | |  |
| 2016 | 0.99 | 0.73 | - | 1.35 | |  |
|  |  |  |  |  | |  |
| **Age group (years)** |  |  |  | |  | 0.07 |
| <60 | 1 |  |  | |  |  |
| 60-70 | 1.10 | 0.97 | - | | 1.24 |  |
| >70 | 1.16 | 0.99 | - | | 1.34 |  |
|  |  |  |  | |  |  |
| **Number of comorbidities (RCS Charlson score)** | | | | |  | <0.01 |
| 0 | 1 |  |  | |  |  |
| 1 | 1.33 | 1.13 | - | | 1.56 |  |
| ≥2 | 1.37 | 0.90 | - | | 2.09 |  |
|  |  |  |  | |  |  |
| **Deprivation status (national quintiles)** | |  |  | |  | 0.30 |
| 1 (least deprived) | 1 |  |  | |  |  |
| 2 | 0.97 | 0.80 | - | | 1.18 |  |
| 3 | 1.00 | 0.83 | - | | 1.20 |  |
| 4 | 0.84 | 0.65 | - | | 1.09 |  |
| 5 (most deprived) | 0.82 | 0.66 | - | | 1.01 |  |
|  |  |  |  | |  |  |
| **Gleason score** |  |  |  | |  |  |
| **6** | 1 |  |  | |  | 0.12 |
| 7 | 1.00 | 0.81 | - | | 1.23 |  |
| ≥8 | 1.16 | 0.95 | - | | 1.41 |  |
|  |  |  |  | |  |  |
| **T-stage** |  |  |  | |  | 0.35 |
| 1 | 1 |  |  | |  |  |
| 2 | 0.87 | 0.64 | - | | 1.19 |  |
| 3 | 0.79 | 0.58 | - | | 1.08 |  |
| 4 | 0.60 | 0.23 | - | | 1.53 |  |
|  |  |  |  | |  |  |
| **N-stage** |  |  |  | |  | 0.95 |
| 0 | 1 |  |  | |  |  |
| 1 | 1.01 | 0.75 | - | | 1.37 |  |
|  |  |  |  | |  |  |
| **GI procedure 1 yr prior to radiotherapy** | | | | | | 0.35 |
| No | 1 |  |  | |  |  |
| Yes | 1.18 | 0.84 | - | | 1.65 |  |
|  |  |  |  | |  |  |
| **Type of radical prostatectomy** | | | | | | 0.89 |
| Robotic | 1 |  |  | |  |  |
| Laparoscopic | 1.05 | 0.84 | - | | 1.31 |  |
| Open | 1.02 | 0.84 | - | | 1.24 |  |
|  |  |  |  | |  |  |
| **Pelvic lymphadenectomy performed** | | | | | | 0.10 |
| No | 1 |  |  | |  |  |
| Yes | 0.88 | 0.75 | - | | 1.03 |  |
|  |  |  |  | |  |  |
| **RT technique** |  |  |  | |  | 0.35 |
| 3D conformal | 1 |  |  | |  |  |
| IMRT | 0.90 | 0.71 | - | | 1.13 |  |
|  |  |  |  | |  |  |
| **Type of RT regimen** |  |  |  | |  | 0.02 |
| Standard | 1 |  |  | |  |  |
| Hypofractionated | 0.68 | 0.49 | - | | 0.94 |  |
|  |  |  |  | |  |  |
| **Time between radical prostatectomy and RT** | | | | | | 0.19 |
| <6 months | 1 |  |  | |  |  |
| ≥6 months | 1.12 | 0.95 | - | | 1.32 |  |

^*^sHR: subdistribution hazard ratios

**Table 2.** Multivariable analysis of factors associated with developing genitourinary (GU) toxicity following prostate bed and pelvic lymph node radiotherapy (PBPLN).

|  | **Adjusted sHR^*^** | **95% Confidence Interval** |  | |  | ***p*** |
| --- | --- | --- | --- | --- | --- | --- |
| **Radiotherapy treatment region** | |  |  | |  | 0.09 |
| PBO | 1 |  |  | |  |  |
| PBPLN | 1.19 | 0.99 | - | | 1.44 |  |
|  |  |  |  | |  |  |
| **Treatment year** |  |  |  | |  | <0.01 |
| 2010 | 1 |  |  |  | |  |
| 2011 | 1.14 | 0.93 | - | 1.41 | |  |
| 2012 | 0.88 | 0.72 | - | 1.08 | |  |
| 2013 | 0.75 | 0.56 | - | 1.01 | |  |
| 2014 | 0.75 | 0.60 | - | 0.92 | |  |
| 2015 | 0.75 | 0.55 | - | 1.02 | |  |
| 2016 | 0.85 | 0.60 | - | 1.18 | |  |
|  |  |  |  |  | |  |
| **Age group (years)** |  |  |  | |  | 0.08 |
| <60 | 1 |  |  | |  |  |
| 60-70 | 0.86 | 0.76 | - | | 0.98 |  |
| >70 | 0.91 | 0.75 | - | | 1.09 |  |
|  |  |  |  | |  |  |
| **Number of comorbidities (RCS Charlson score)** | | | | |  | <0.01 |
| 0 | 1 |  |  | |  |  |
| 1 | 1.33 | 1.16 | - | | 1.53 |  |
| ≥2 | 1.49 | 0.98 | - | | 2.28 |  |
|  |  |  |  | |  |  |
| **Deprivation status (national quintiles)** | |  |  | |  | 0.04 |
| 1 (least deprived) | 1 |  |  | |  |  |
| 2 | 1.06 | 0.85 | - | | 1.33 |  |
| 3 | 0.91 | 0.73 | - | | 1.15 |  |
| 4 | 1.19 | 0.96 | - | | 1.48 |  |
| 5 (most deprived) | 1.31 | 1.05 | - | | 1.64 |  |
|  |  |  |  | |  |  |
| **Gleason score** |  |  |  | |  |  |
| **6** | 1 |  |  | |  | 0.12 |
| 7 | 1.19 | 0.99 | - | | 1.45 |  |
| ≥8 | 1.23 | 1.00 | - | | 1.50 |  |
|  |  |  |  | |  |  |
| **T-stage** |  |  |  | |  | 0.19 |
| 1 | 1 |  |  | |  |  |
| 2 | 0.73 | 0.54 | - | | 1.0 |  |
| 3 | 0.80 | 0.61 | - | | 1.06 |  |
| 4 | 1.06 | 0.50 | - | | 2.23 |  |
|  |  |  |  | |  |  |
| **N-stage** |  |  |  | |  | 0.72 |
| 0 | 1 |  |  | |  |  |
| 1 | 0.95 | 0.73 | - | | 1.24 |  |
|  |  |  |  | |  |  |
| **GU procedure 1 yr prior to radiotherapy** | | | | | | <0.01 |
| No | 1 |  |  | |  |  |
| Yes | 1.43 | 1.11 | - | | 1.88 |  |
|  |  |  |  | |  |  |
| **Type of radical prostatectomy** | | | | | | 0.26 |
| Robotic | 1 |  |  | |  |  |
| Laparoscopic | 1.05 | 0.82 | - | | 1.35 |  |
| Open | 1.17 | 0.93 | - | | 1.47 |  |
|  |  |  |  | |  |  |
| **Pelvic lymphadenectomy performed** | | | | | | 0.55 |
| No | 1 |  |  | |  |  |
| Yes | 1.04 | 0.90 | - | | 1.21 |  |
|  |  |  |  | |  |  |
| **RT technique** |  |  |  | |  | 0.10 |
| 3D conformal | 1 |  |  | |  |  |
| IMRT | 0.84 | 0.69 | - | | 1.03 |  |
|  |  |  |  | |  |  |
| **Type of RT regimen** |  |  |  | |  | 0.83 |
| Standard | 1 |  |  | |  |  |
| Hypofractionated | 0.98 | 0.78 | - | | 1.21 |  |
|  |  |  |  | |  |  |
| **Time between radical prostatectomy and RT** | | | | | | 0.11 |
| <6 months | 1 |  |  | |  |  |
| ≥6 months | 0.86 | 0.71 | - | | 1.04 |  |

^*^sHR: subdistribution hazard ratios
